# Antimicrobial resistance awareness and associated factors among family physicians in Turkey: a national cross-sectional study

**DOI:** 10.1186/s12875-026-03444-w

**Published:** 2026-06-25

**Authors:** Zeynep Sedef Varol, İlkay Akbulut, Emrah Kırımlı, Buğu Usanma Koban, Aysel Başer, Zeynep Sofuoğlu, Sabri Atalay

**Affiliations:** 1 Department of Public Health, Faculty of Medicine, İzmir Democracy University, İzmir, Turkey; 2 Department of Infectious Diseases and Clinical Microbiology, Tepecik Training and Research Hospital, İzmir, Turkey; 3 İstanbul Provincial Health Directorate, İstanbul, Turkey; 4 Department of Family Medicine, Marmara University Faculty of Medicine, İstanbul, Turkey; 5 Department of Medical Education, Faculty of Medicine, İzmir Democracy University, İzmir, Turkey

**Keywords:** Antimicrobial resistance, Antimicrobial stewardship, Primary care, Health systems, Policy implementation, Rational drug use

## Abstract

**Background:**

Antimicrobial resistance (AMR) is a major global public health threat requiring effective antimicrobial stewardship across healthcare systems. Primary care physicians are responsible for a substantial proportion of antibiotic prescribing; however, evidence on awareness of antimicrobial stewardship policies in primary care remains limited, particularly in middle-income settings. This study aimed to assess antimicrobial resistance awareness among family physicians and to examine its association with exposure to national policy initiatives.

**Methods:**

A nationwide cross-sectional survey was conducted between October 2025 and January 2026 among 351 family physicians across Turkey using a stratified quota-based convenience sampling approach. AMR awareness was measured using the Turkish-adapted 23-item Antibiotic Resistance Awareness Scale, originally developed using Rasch Measurement Theory. Internal consistency was assessed using Cronbach’s alpha. Associations between AMR awareness scores and sociodemographic, educational, and contextual factors—including specialist status, sources of AMR-related information, perceived adequacy of AMR knowledge for daily practice, and awareness of national rational drug use policies—were examined using multivariable linear regression analysis.

**Results:**

The mean standardized AMR awareness score was 76.55 ± 14.95 (Cronbach’s α = 0.934). AMR awareness scores were not significantly associated with age, gender, or years of professional experience (*p* > 0.05). Higher AMR awareness scores were independently associated with perceived adequacy of AMR-related knowledge for daily clinical practice (*p* < 0.001) and awareness of national rational drug use policies (B = 4.28, *p* = 0.010). Demographic characteristics were not independently associated with AMR awareness scores.

**Conclusions:**

Antimicrobial stewardship–related competencies in primary care were more strongly associated with policy exposure and knowledge, training, and institutional awareness reinforced through routine clinical practice than with demographic factors. Strengthening policy-linked and practice-oriented educational strategies may support AMR awareness initiatives in primary care settings. These findings may contribute to future research and policy discussions regarding antimicrobial stewardship awareness and related educational initiatives in primary care settings.

**Supplementary Information:**

The online version contains supplementary material available at https://doi.org/10.1186/s12875-026-03444-w.

## Introduction

Antimicrobial resistance (AMR) is a global public health crisis, causing preventable deaths, disabilities, health system strain, and rising costs [1]. Reports from the World Health Organization’s (WHO) Global Antimicrobial Resistance and Use Surveillance System (GLASS) show that AMR is an increasing worldwide burden, responsible for about five million deaths in 2019, with over 1.2 million directly attributable to AMR [2].

A major driver of AMR is the inappropriate and excessive use of antibiotics [3]. Thus, healthcare professionals’ knowledge and clinical decisions are central to antimicrobial stewardship [4, 5]. Awareness of AMR is considered an important precursor to antimicrobial stewardship behavior, as clinicians’ perceptions and understanding of resistance may influence prescribing practices, adherence to treatment guidelines, and engagement with stewardship interventions [5, 6].

The WHO’s Global Action Plan on Antimicrobial Resistance prioritizes raising awareness among health workers and the public and advocates for a multisectoral One Health approach [7]. Within this framework, the One Health perspective emphasizes the interconnected roles of human, animal, and environmental health in the emergence and spread of antimicrobial resistance, highlighting the need for integrated stewardship and awareness strategies across sectors [7, 8].

Despite increasing international attention to AMR, reliable tools for assessing awareness and stewardship-related competencies in clinical settings remain limited [8]. Many previous studies have relied on locally developed questionnaires with limited comparability across settings, making systematic assessment and monitoring difficult [9]. The Antibiotic Resistance Awareness Scale, developed by Pinto Jimenez et al., was designed to provide a multidimensional and theoretically grounded assessment framework for evaluating AMR awareness among healthcare professionals, though its use among family physicians in Turkey has been limited [10, 11].

Primary care, where most antibiotics are prescribed, is a key setting for AMR. Factors such as diagnostic uncertainty and patient expectations influence antibiotic use, and family physicians’ awareness shapes both individual and community prescribing patterns [12, 13]. Therefore, assessing AMR awareness in primary care may help identify stewardship-related educational needs and policy implementation gaps at the health system level.

This study aimed to assess AMR awareness among family physicians in Turkey using the Turkish-adapted version of the Antibiotic Resistance Awareness Scale and to explore factors associated with AMR awareness in a nationwide primary care sample.

## Methods

### Study design

This study was designed as a nationwide cross-sectional analytical online survey conducted among family physicians working in Turkey to evaluate antimicrobial resistance (AMR) awareness. The study also included a Turkish language adaptation and internal consistency assessment component of the Antibiotic Resistance Awareness Scale. The present study did not aim to perform a full psychometric validation of the Turkish version of the scale.

### Measurement ınstrument

Awareness of antimicrobial resistance was assessed using the Antibiotic Resistance Awareness Scale (ABR Awareness Scale), developed and previously evaluated using Rasch Measurement Theory by Pinto Jimenez et al. (2023). The questionnaire consisted of the previously published 23-item Antibiotic Resistance Awareness Scale together with additional author-developed questions regarding demographic characteristics, educational exposure, and policy-related contextual factors. This 23-item scale, grounded in Rasch Measurement Theory, evaluates four key domains: biological mechanisms of antibiotic resistance, the role of antibiotic use in resistance development, transmission and infection control, and the recognition and identification of resistance [10].

Responses are recorded on a four-point Likert scale (strongly agree, agree, disagree, strongly disagree). To address disordered thresholds identified in the original validation, Rasch model–based rescoring was applied to specific items (1, 2, 4, 5, 14, 17, and 23) in line with the original scoring guidelines. After rescoring, standardized scores from 0 to 100 were generated, with higher scores reflecting greater awareness. In the original validation study, reliability was assessed using the Person Separation Index (PSI), with a reported value of 0.88 [10, 11].

The developers advise that the scale is best suited for examining contextual variation within countries, rather than for direct cross-national comparisons. Consistent with this, the present study prioritized geographic diversity in sampling but did not conduct formal region-level comparisons. For descriptive and comparative analyses, raw scores were linearly transformed to a 0–100 scale, as recommended in previous studies [10, 11]. For transparency, the Turkish-language questionnaire used for data collection is provided as Additional File 2.

### Translation and cultural adaptation

The Turkish adaptation of the scale was carried out using a forward–backward translation method. The initial translation was performed by a medical doctor with university-level language training, and semantic equivalence with the original scale was ensured through back-translation. The linguistic and content validity of the Turkish version was evaluated by a panel of nine experts (three infectious disease specialists, one epidemiologist, two public health specialists, one pharmacology specialist, and two family medicine specialists) using the Davis [14] method (1992). The scale-level content validity index (S-CVI/Ave) was found to be 0.95. Based on expert feedback, only minor linguistic adjustments were made to strengthen semantic integrity, while the conceptual structure of the scale was preserved.

### Variables

Independent variables were grouped into four categories according to factors identified in the literature as potentially associated with AMR awareness. These included sociodemographic and professional characteristics (age, gender, year of graduation, professional experience, years in family medicine practice, specialist status, and region of employment); education and information sources (methods of obtaining information related to AMR or antimicrobial stewardship and participation in specific training activities); perceived educational adequacy variables (participants’ self-reported evaluations regarding the adequacy of AMR-related education during formal medical training and for routine clinical practice); and contextual or policy-related factors (perceived prioritization of AMR relative to other health problems in the workplace and awareness of the Ministry of Health’s rational drug use initiatives).

Information-source diversity was dichotomized as reliance on a single source versus multiple sources of AMR-related information. Awareness of national rational drug use policies referred to self-reported awareness of Ministry of Health initiatives related to rational antibiotic use and antimicrobial stewardship practices.

### Sampling and data collection

The target population consisted of approximately 24,000 family physicians in Turkey. Using conservative assumptions (*p* = 0.50, 95% confidence level, ± 5% margin of error), the minimum required sample size was calculated as approximately 370 participants. A total of 351 family physicians participated in the study, corresponding to a margin of error of approximately ± 5.1%.

A stratified quota-based convenience sampling approach was used. Sampling quotas were determined according to the population distribution of Turkey’s NUTS-1 regions to improve geographic representation across the country.

The study population consisted of physicians actively working in family medicine practice in Turkey during the study period. Eligible participants were family physicians who voluntarily agreed to participate in the online survey. Physicians who were not actively practicing family medicine were excluded from the study.

Data were collected online between October 25, 2025, and January 5, 2026, through an open online survey distributed via provincial medical chambers, professional associations, and physician professional networks. Recruitment was conducted across all NUTS-1 regions of Turkey using multiple professional communication channels to improve geographic diversity and reduce the risk of recruitment through a single professional network. Regional data collection continued until predefined quota targets were reached.

Family physicians were classified by province and aggregated into three meta-regions (Western, Central, and Eastern Turkey) to evaluate regional distribution patterns. Although Western Turkey was slightly overrepresented and Central and Eastern regions were relatively underrepresented, the sample included participants from all major geographic regions of the country (see Supplementary Table 1).

Participation was voluntary and anonymous. No personally identifiable information was collected. Participants were electronically informed about the study objectives, confidentiality principles, voluntary participation, and their right to withdraw before survey completion. Submission of the online questionnaire was accepted as provision of informed consent.

As the survey was distributed through open professional networks, the exact response rate could not be calculated, and duplicate participation could not be fully excluded. However, nationwide recruitment strategies and regional quota-based sampling were used to improve geographic diversity and broaden participant representation. All submitted questionnaires were complete and were included in the final analysis.

### Statistics

Internal consistency of the scale was assessed using Cronbach’s alpha, and corrected item–total correlations were examined to evaluate item performance. Descriptive analyses were performed for participant characteristics and questionnaire responses. Comparisons between groups were conducted using Student’s t-test for approximately normally distributed variables and the Kruskal–Wallis test for non-normally distributed variables.

For descriptive subgroup comparisons presented in Table 3, age, professional experience, and years working as a family physician were categorized using cutoffs that approximated the central tendency of the distributions (40 years, 15 years, and 10 years, respectively) to facilitate interpretation and presentation of subgroup comparisons. These categorizations were used only for descriptive subgroup analyses and were not included in the multivariable regression analyses.

Multivariable linear regression analysis was performed to explore associations between AMR awareness scores and selected participant characteristics, educational variables, and policy-related contextual factors. Variables included in the multivariable model were selected a priori based on conceptual relevance, previous literature examining factors associated with AMR awareness and antimicrobial stewardship [6, 10], and the objectives of the present study rather than solely on statistical significance in univariable analyses. Candidate variables considered for multivariable analysis included family medicine specialist status, diversity of AMR information/training sources, perceived adequacy of AMR education/training during undergraduate or graduate education, perceived adequacy of AMR knowledge/training for daily practice, and awareness of national rational drug use policies. All candidate variables were retained in the final multivariable model.

Categorical predictors were entered using binary indicator coding. Reference categories for the regression analyses were specialist physician, single AMR information/training source, adequate AMR education/training during undergraduate or graduate education, sufficient AMR knowledge/training for daily practice, and partial/no awareness of national rational drug use policies.

The multivariable analyses were intended to explore potential associations with AMR awareness scores rather than to identify causal determinants or predictors. Model assumptions were assessed using residual diagnostics and collinearity statistics. Visual inspection of residual plots indicated no substantial departures from linearity or homoscedasticity. Standardized residuals ranged from − 2.62 to 2.22, suggesting no substantial outliers. Variance inflation factor (VIF) values ranged from 1.02 to 1.62, and the maximum condition index was 20.15, indicating no evidence of problematic multicollinearity. The maximum Cook’s distance value was 0.054, suggesting no single observation exerted undue influence on the regression model.

Statistical analyses were performed using IBM SPSS Statistics for Windows, Version 28.0 (IBM Corp., Armonk, NY, USA). A two-sided *p*-value < 0.05 was considered statistically significant.

### Ethical considerations

Ethics approval for the study was obtained from the Buca Seyfi Demirsoy Training and Research Hospital Non-Interventional Research Ethics Committee (Decision No. 2025/516). Permission to use the scale was obtained from the scale’s original developers. The study was conducted in accordance with the Declaration of Helsinki. Participation was voluntary and informed consent was obtained electronically from all participants prior to survey completion.

## Results

### Scale performance

The scale demonstrated excellent internal consistency (Cronbach’s α = 0.934). Corrected item–total correlations ranged from 0.46 to 0.71, and removal of any item did not improve internal consistency, supporting the retention of all 23 items (see Supplementary Table 2).

The mean standardized score was calculated as 76.55 ± 14.95 (range: 27.42–100.0). The median standardized AMR awareness score was 75.81 (IQR: 25.81), with scores distributed across a broad range and without substantial clustering at the maximum score level.

### Descriptive and exploratory findings

The mean age of participants was 42.4 ± 11.3 years (range: 25–70), and 55.3% (*n* = 194) were male. The average duration of professional experience was 16.2 ± 11.4 years, while the average time working as a family physician was 9.3 ± 6.5 years. Of the family physicians, 47.0% were specialists in family medicine, while 53.0% had not received specialty training (Table 1).

**Table 1:** Participant characteristics, AMR-related education, and contextual perceptions (*n* = 351)

Variable	Value^*^	% / Range
Age (years)	42.4 ± 11.3	25–70
Years of professional experience	16.2 ± 11.4	1–45
Years working as a family physician	9.3 ± 6.5	1–39
Sex		
Female	157	44.7**%**
Male	194	55.3**%**
Family medicine specialist		
Yes	165	47.0**%**
No	186	53.0**%**
Source of AMR knowledge		
Single source	36	10.3**%**
Multiple sources	315	89.7**%**
AMR adequately covered during formal training curriculum		
Agree / Strongly agree	230	65.5**%**
Disagree / Strongly disagree	121	34.5**%**
Current AMR knowledge sufficient for daily practice		
Agree / Strongly agree	236	67.2**%**
Disagree / Strongly disagree	115	32.8**%**
Attended specific training on AMR and/or antimicrobial stewardship		
Yes	106	30.2**%**
No	245	69.8**%**

*Continuous variables are presented as mean ± standard deviation and range; categorical variables are presented as *n* (%)

The sample included family physicians from all major geographic regions of Turkey and reflected diversity in demographic and professional characteristics.

Family physicians’ main sources of information on antimicrobial resistance were university education and in-service training. However, congresses and scientific publications also represented important sources. Although informal learning and online training were notable, participation in training provided by international organizations was limited (Supplementary Table 3). The vast majority of participants (89.7%) reported obtaining information about antimicrobial resistance from multiple sources (Table 1).

A total of 65.5% of participants believed that sufficient information on antimicrobial resistance was provided during their undergraduate or residency education, and 67.2% considered their current knowledge and training on antimicrobial resistance to be adequate for daily clinical practice. In contrast, only 30.2% reported having attended specific training on antimicrobial resistance and/or antimicrobial stewardship. This pattern may suggest that participants relied predominantly on formal medical education and routine clinical experience rather than structured continuing stewardship-focused training programs (Table 1).

Among participants, 31.9% agreed with the statement that a patient’s health could deteriorate if antibiotics were not prescribed. When comparing antibiotic resistance to other health issues in their work environment, 60.7% rated malnutrition, 71.8% rated chronic diseases, and 58.7% rated hygiene and sanitation problems as more important health concerns than antibiotic resistance. The proportion of those who considered other infectious diseases a higher priority was 45.6%, while 33.6% considered trauma and accidents to be more important (Table 2).

**Table 2:** Contextual perceptions related to antibiotic use and national policies

Contextual statement / policy-related factor	Agree / Strongly agree *n* (%)	Disagree / Strongly disagree *n* (%)
1. If I do not prescribe/dispense an antibiotic, there could be a worse health outcome for the person I am treating	112 (31.9%)	239 (68.1%)
2. At my place of work, I consider malnutrition a higher concern than antibiotic resistance	213 (60.7%)	138 (39.3%)
3. At my place of work, I consider chronic disease a higher concern than antibiotic resistance	252 (71.8%)	99 (28.2%)
4. At my place of work, I consider the level of hygiene and sanitation a higher concern than antibiotic resistance	206 (58.7%)	145 (41.3%)
5. At my place of work, I consider other infectious diseases a higher concern than antibiotic resistance	160 (45.6%)	191 (54.4%)
6. At my place of work, I consider trauma and accidents (for example, traffic accidents and burns) a higher concern than antibiotic resistance	118 (33.6%)	233 (66.4%)
Change in prescribing behavior after Ministry of Health performance policy		
Prescribe fewer antibiotics	90 (25.6%)	—
No change	261 (74.4%)	—
Awareness of national rational drug use policies		
Yes	230 (65.5%)	—
Partly	110 (31.3%)	—
No	11 (3.1%)	—
Belief that performance-based prescribing policies can reduce AMR		
Yes	111 (31.6%)	—
No opinion	27 (7.7%)	—
No	213 (60.7%)	—

All values are presented as *n* (%). Contextual perception items (1–6) are reported in accordance with the original Antibiotic Resistance Awareness Scale framework and presented as agree/strongly agree versus disagree/strongly disagree. For variables with more than two response categories (awareness of national rational drug use policies; belief that performance-based prescribing policies can reduce AMR), all response options are presented separately

A total of 65.5% of family physicians reported being aware of the Ministry of Health’s rational drug use initiatives, while 31.3% were partially aware and 3.1% were unaware of these programs. Only 31.6% of participants believed that performance-based practices could reduce antibiotic resistance. A total of 25.6% reported prescribing fewer antibiotics after the Ministry of Health’s performance-based policy on antibiotic prescribing (Table 2).

Mean AMR awareness scores did not differ significantly by age group, sex, or duration of professional experience (Table 3). Similarly, no statistically significant differences were observed according to years worked as a family physician. In contrast, higher awareness scores were observed among family physicians with specialist training compared to non-specialists (*p* = 0.034). Participants who reported exposure to multiple sources of AMR-related information also demonstrated higher scores than those relying on a single source (*p* = 0.041).

**Table 3:** Exploratory comparisons of mean AMR awareness scores across selected participant characteristics (*n* = 351)

Variable	Category	AMR awareness score^†^	*p* value*
Age group	≤40 years (*n* = 187)	76.66 ± 15.11	0.879
	>40 years (*n* = 164)	76.42 ± 14.81	
Sex	Female	75.29 ± 13.97	0.158
	Male	77.56 ± 15.67	
Years of professional experience	≤15 years	76.59 ± 14.98	0.948
	>15 years	76.49 ± 14.97	
Years working as a family physician	≤10 years	76.50 ± 15.15	0.951
	>10 years	76.60 ± 14.74	
Family medicine specialization	Yes	78.34 ± 14.27	**0.034**
	No	74.96 ± 15.40	
Sources of AMR training/information	Single source	71.73 ± 17.28	**0.041**
	Multiple sources	77.10 ± 14.59	
AMR covered during undergraduate/graduate curriculum	Agree	77.98 ± 15.21	**0.013**
	Disagree	73.82 ± 14.13	
AMR knowledge sufficient for daily practice	Agree	79.06 ± 14.54	**< 0.001**
	Disagree	71.39 ± 14.52	
Participation in AMR/AMS-specific training	Yes	78.71 ± 15.23	0.074
	No	75.61 ± 14.76	

*Independent samples t-test was used for two-group comparisons with approximately normal distribution

† Data are presented as mean ± standard deviation

Values shown in bold indicate statistical significance (*p* < 0.05)

Perceived adequacy of AMR education was consistently associated with higher awareness scores. Participants who agreed that AMR had been sufficiently covered during their formal education scored higher than those who disagreed (*p* = 0.013). Likewise, those who considered their current AMR knowledge sufficient for daily clinical practice had substantially higher scores (*p* < 0.001). Although participants who had attended AMR- or antimicrobial stewardship–specific training tended to have higher scores, this difference did not reach statistical significance (*p* = 0.074) (Table 3).

AMR awareness scores according to contextual priorities and Ministry of Health–related factors are presented in Table 4. AMR awareness scores did not differ significantly across contextual priority items reflecting competing health concerns in the workplace (Items 1–6; all *p* > 0.05). No significant difference was observed according to self-reported changes in prescribing behavior following the Ministry of Health’s performance-based policy. In contrast, awareness of national rational drug use policies was associated with higher AMR awareness scores (*p* < 0.001), whereas beliefs regarding the effectiveness of performance-based prescribing policies in reducing AMR were not associated with awareness scores.

**Table 4:** AMR awareness scores according to contextual priorities and Ministry of Health–related factors

Variable	Category	Scale score*	*p* value ^a, b^
Contextual perception items (Items 1–6)			
Item 1	Disagree	76.12 ± 15.10	0.433 ᵃ
	Agree	77.46 ± 14.66	
Item 2	Disagree	76.54 ± 14.91	0.997 ᵃ
	Agree	76.55 ± 15.01	
Item 3	Disagree	76.39 ± 15.19	0.904 ᵃ
	Agree	76.61 ± 14.89	
Item 4	Disagree	76.83 ± 15.59	0.766 ᵃ
	Agree	76.35 ± 14.52	
Item 5	Disagree	77.23 ± 14.84	0.348 ᵃ
	Agree	75.73 ± 15.10	
Item 6	Disagree	75.93 ± 14.92	0.279 ᵃ
	Agree	77.76 ± 15.01	
Ministry of Health–related factors			
Change in prescribing behavior after Ministry of Health performance policy	Prescribe fewer antibiotics	74.84 ± 15.02	0.209 ᵃ
	No change	77.14 ± 14.91	
Awareness of national rational drug use policies	Yes	79.03 ± 17.42	**< 0.001** ^**a**^
	Partly/No	72.79 ± 14.39	
Belief that performance-based prescribing policies can reduce AMR	Yes	72.58 (25.81)	0.711^b^
	No opinion	75.81 (20.97)	
	No	79.03 (27.42)	

a Independent samples t-test was used for two-group comparisons with approximately normal distribution

b Kruskal–Wallis test was used for comparisons involving three groups; where applicable, post-hoc pairwise comparisons were conducted using the Mann–Whitney U test

* Scale score is presented as mean ± standard deviation for parametric comparisons and as median (interquartile range) for non-parametric comparisons, as appropriate

Values shown in bold indicate statistical significance (*p* < 0.05)

In multivariable linear regression analysis, awareness of national rational drug use policies remained independently associated with higher AMR awareness scores (B = 4.28, *p* = 0.010). In contrast, perceiving AMR knowledge and training as insufficient for daily practice was associated with significantly lower awareness scores (B = − 6.61, *p* = 0.002) (Table 5).

**Table 5:** Multivariable linear regression analysis of factors associated with AMR awareness score (*n* = 351)

Predictor	B (Unstandardized)	SE	β (Standardized)	*p* value
Constant	67.25	5.80	—	**< 0.001**
Non-specialist physician (reference: specialist physician)	−2.84	1.55	−0.10	0.068
Multiple AMR information/training sources (reference: single source)	3.01	2.59	0.06	0.246
Perceived inadequacy of AMR education/training during undergraduate or graduate education (reference: adequate education/training)	0.55	2.02	0.02	0.787
Perceived insufficiency of AMR knowledge/training for daily practice (reference: sufficient knowledge/training)	−6.61	2.08	−0.21	**0.002**
Awareness of national rational drug use policies (reference: partial/no awareness)	4.28	1.66	0.14	**0.010**

Model fit statistics: R = 0.300; R² = 0.090; Adjusted R² = 0.077; F(5,345) = 6.82, *p* < 0.001

Dependent variable: AMR awareness score (range 0–100; higher scores indicate higher awareness). *B* unstandardized regression coefficient, *SE* standard error, β standardized regression coefficient

Values shown in bold indicate statistical significance (*p* < 0.05)

## Discussion

This nationwide study provides one of the first national assessments of antimicrobial resistance awareness among primary care physicians in a middle-income health system using a Turkish-adapted instrument with internal consistency assessment. Rather than differing according to demographic characteristics, AMR awareness scores were more strongly associated with perceived adequacy of knowledge and training reinforced through routine clinical practice, as well as awareness of national policy initiatives. This interpretation is also supported by stewardship-oriented implementation studies from middle-income settings indicating that institutionally supported educational strategies and continuous monitoring approaches may contribute to improvements in antimicrobial use practices in clinical care [15]. These findings support the possibility that institutional and policy-related contexts may play an important role in AMR awareness within primary care settings.

Although awareness of core AMR concepts was high, knowledge alone may not fully capture preparedness for antimicrobial stewardship practices in primary care. In our study, AMR awareness scores were more strongly associated with perceived adequacy of training and awareness of national rational drug use policies than with demographic characteristics. This pattern suggests that institutional exposure and policy engagement may be associated with differences in stewardship-related competencies in primary care settings. However, because perceived adequacy variables were self-reported, the observed associations may also partially reflect self-perception bias or greater confidence in AMR-related knowledge rather than objectively measured competency.

The Antibiotic Resistance Awareness Scale, originally developed using Rasch Measurement Theory, enabled a structured assessment of AMR awareness domains in the present study. As recommended by the original authors, cross-country score comparisons were avoided; instead, emphasis was placed on domain-level patterns and factors associated with higher awareness scores [10]. This approach aligns with the growing recognition that AMR control requires context-sensitive system assessments rather than simple numerical benchmarking.

In our study, participants demonstrated high levels of agreement with several core AMR concepts. Most participants demonstrated agreement with key AMR-related concepts such as the ability of microorganisms to acquire resistance through mutation, the status of antimicrobial resistance as a global health threat, and the importance of infection control measures. This finding is consistent with results from a study conducted by Kuttikkunnummal and colleagues in Kerala, India, which used the same scale and similarly reported high levels of awareness among physicians regarding fundamental AMR mechanisms [16].

In contrast, items addressing the environmental and animal health dimensions of the One Health framework received lower scores in our sample. In particular, awareness was relatively low regarding the use of antibiotics intended for humans in animal treatment, the entry of antibiotics into sewage systems, and their release into the environment. Comparable knowledge gaps were also reported in the Kerala study, especially in more complex domains such as resistance mechanisms, surveillance, and antibiotic optimization [16]. Furthermore, a study by Karasneh et al. in Lebanon found that while approximately 60% of physicians recognized the contribution of antibiotic use in livestock and food production to AMR, only 32% acknowledged the role of environmental wastewater [17]. Taken together, these findings suggest that AMR may be perceived predominantly as a clinical and human-centered issue, while environmental and animal health dimensions appear to be less fully integrated into physicians’ conceptual frameworks. Preventive dimensions of antimicrobial stewardship may also be relatively under-recognized in primary care settings, despite the important role of vaccination and infection prevention measures in reducing unnecessary antimicrobial use and limiting AMR emergence [18]. These findings highlight the potential need to strengthen environmental and animal health dimensions of AMR within undergraduate medical education, continuing professional training, and broader stewardship-oriented public health initiatives.

In this study, only approximately one-third of family physicians expressed concern that a patient’s health might deteriorate if antibiotics were not prescribed. This finding suggests that the concern about “worse clinical outcomes” reported in multi-country scale development studies by Pinto Jimenez et al., particularly in low- and middle-income countries may be less pronounced in the Turkish primary care context. This difference may be attributable to structural aspects of family medicine practice in Turkey [6, 19]. Indeed, studies from Egypt, Lebanon, and Ghana have also indicated that AMR awareness is more closely linked to the healthcare system context and clinical priorities than to demographic characteristics [17, 20, 21].

The fact that participants rated chronic diseases, malnutrition, and hygiene–sanitation issues as higher priorities than antibiotic resistance indicates that AMR is perceived as a long-term, system-level threat. This supports the notion that AMR awareness may be associated not only with individual knowledge, but also with clinical risk perception and the broader healthcare system context [6, 22–24].

In our study, demographic variables such as age, gender, and years of professional experience were not significantly associated with AMR awareness levels, which is consistent with findings in the literature. For example, in a hospital-based study of healthcare workers in Egypt, Khalaf and colleagues found no association between AMR awareness and either gender or years of professional experience [20]. Similarly, studies from Ghana and Lebanon also reported that age and gender were not significantly associated with AMR knowledge or awareness [17, 21]. These results suggest that AMR awareness may be more closely associated with professional education and institutional exposure than with demographic characteristics.

The multivariable analyses identified several factors independently associated with AMR awareness scores. Perceived adequacy of knowledge and training on antimicrobial resistance for daily clinical practice was independently associated with higher AMR awareness scores. By contrast, inclusion of AMR as part of the formal educational curriculum did not show an independent association with awareness scores once other variables were accounted for in the model. This suggests that awareness levels may be related not only to the presence of knowledge, but also to the extent to which such knowledge is reinforced through routine clinical practice [6, 25]. Similarly, Bahamdan et al. from Saudi Arabia reported that healthcare professionals who participated in ongoing and repeated AMR training, including online courses, demonstrated higher levels of knowledge and more positive attitudes [26].

In this study, the finding that awareness of the Ministry of Health’s rational drug use initiatives was associated with higher AMR awareness scores suggests that AMR awareness may be associated not only with individual knowledge, but also with health policies and institutional frameworks [23]. This aligns with findings from Egypt and other middle-income settings, where awareness of rational drug use policies has been associated with higher AMR awareness levels [20]. The World Health Organization’s inclusion of rational drug use as a core component of its antimicrobial resistance strategies, and the prioritization of rational antibiotic use in Turkey’s primary care policies, further support this relationship [19, 27]. On the other hand, the lack of a significant association between belief in the effectiveness of performance-based prescribing policies and AMR awareness scores indicates that structural and regulatory interventions alone may not be sufficient to increase awareness [28]. In primary care settings, stewardship-oriented continuing education, audit and feedback mechanisms, and integration of rational prescribing principles into routine clinical workflows may represent relevant institutional strategies for strengthening AMR awareness.

The findings suggest that AMR awareness scores were more closely associated with practice-oriented knowledge, training, and institutional awareness than with demographic factors. Awareness of rational drug use initiatives and practice-oriented educational exposure were independently associated with higher AMR awareness scores. In contrast, awareness of the environmental and animal health dimensions of the One Health approach remains limited, highlighting the need to reinforce these areas in primary care education and policy. These findings support the potential value of policy-supported and practice-oriented educational strategies for strengthening AMR awareness in primary care settings. These findings should be interpreted in light of certain methodological limitations.

This study has several limitations. First, its cross-sectional design does not allow for causal inferences. Additionally, the reliance on self-reported data may have introduced social desirability bias. Because perceived adequacy measures were self-reported, self-perception bias may also have influenced some observed associations. Furthermore, the use of stratified convenience sampling and online recruitment through professional networks may have introduced selection bias and limited representativeness. The study also did not assess actual prescribing behavior or clinical decision-making practices, limiting the ability to directly relate AMR awareness scores to antimicrobial stewardship performance in clinical settings. Nonetheless, the use of a previously developed AMR awareness scale with strong internal consistency and the inclusion of a large sample of family physicians enhance the relevance of the findings for primary care. Comprehensive psychometric evaluation of the Turkish-adapted version of the scale was beyond the scope of the present study and may require further investigation in future validation-focused research. While participation from some geographic regions was relatively low, the overall sample size was sufficient and included diverse regions, supporting the generalizability of the results to primary care settings across Turkey.

Given the cross-sectional design of the study, these findings should be interpreted as exploratory associations that may support future hypothesis-generating research rather than causal relationships.

## Conclusion

AMR awareness among family physicians in Turkey was more strongly associated with perceived adequacy of training and awareness of national rational drug use policies than with demographic characteristics. These findings highlight that practice-oriented education and policy exposure may represent important factors associated with antimicrobial stewardship–related awareness in primary care. Awareness of the environmental and animal health dimensions of the One Health framework remained comparatively limited, suggesting the need for broader stewardship-oriented educational approaches. Strengthening structured, practice-oriented, and policy-linked educational strategies may support improvements in AMR awareness and contribute to more effective antimicrobial stewardship implementation in primary care settings.

## Supplementary Information


Supplementary Material 1.



Supplementary Material 2.


## Data Availability

The datasets generated and/or analyzed during the current study are available from the corresponding author on reasonable request.
