# Supplementary material for: Antimicrobial resistance awareness and associated factors among family physicians in Turkey: a national cross-sectional study
Source: BMC Prim Care. 2026 Jun 25;27:320. doi: 10.1186/s12875-026-03444-w (PMC13523260; doi:10.1186/s12875-026-03444-w)
Supplement: Supplementary file 1 [file 12875_2026_3444_MOESM1_ESM.docx]

**Supplementary Tables**

| Supplementary Table S1.Population-based expected versus observed distribution of the study sample across meta-regions of Turkey (n = 351) | | | | | | | |
| --- | --- | --- | --- | --- | --- | --- | --- |
| Meta-region | NUTS-1 regions included | Population  ≈,million | Expected n* | Expected % | Observed n | Observed % | Relative difference† |
| Western Turkey | TR1, TR2, TR3, TR4 | 39.10 | 161 | 45.9 | 176 | 50.1 | +9.2% overrepresented |
| Central Turkey | TR5, TR6, TR7 | 23.42 | 96 | 27.4 | 86 | 24.5 | −10.5% underrepresented |
| Eastern Turkey | TR8, TR9, TRA, TRB, TRC | 22.94 | 94 | 26.8 | 89 | 25.4 | −5.2% underrepresented |
| Total |  | 85.46 | 351 | 100.0 |  |  |  |
| * Expected numbers were calculated by distributing the total sample size (n = 351) proportionally according to the population size of each meta-region. † Relative difference was calculated as: (Observed % − Expected %) / Expected % × 100. | | | | | | | |

| Supplementary Table S2. Internal consistency and item–total statistics of the Antibiotic Resistance Awareness Scale (n = 351) | | | | |
| --- | --- | --- | --- | --- |
| Item (original wording) | Mean | SD | Corrected Item–Total Correlation | Cronbach’s α if Item Deleted |
| 1. Antibiotic resistance is when a microorganism becomes resistant to antibiotics | 1.50 | 0.51 | 0.61 | 0.93 |
| 2. Some microorganisms can mutate and therefore become resistant to antibiotics | 1.57 | 0.52 | 0.67 | 0.93 |
| 3. Some microorganisms can transfer resistance by exchanging genetic material | 2.57 | 0.51 | 0.71 | 0.93 |
| 4. Antibiotic resistance can develop if antibiotics are given when they are not indicated, for example, when a person has a viral infection | 1.57 | 0.56 | 0.64 | 0.93 |
| 5. Antibiotic resistance can develop if courses of antibiotic treatment are interrupted, for example, stopping and starting again halfway through a prescribed course | 1.59 | 0.55 | 0.64 | 0.93 |
| 6. Antibiotic resistance can develop if antibiotics are given/taken in lower than recommended doses | 2.42 | 0.67 | 0.58 | 0.93 |
| 7. Antibiotic resistance can develop if antibiotics are used to treat bacterial colonisation rather than bacterial infection | 2.18 | 0.78 | 0.48 | 0.93 |
| 8. Antibiotic resistance can develop if antibiotics are used as a ‘just in case’ measure for any routine procedure | 2.10 | 0.73 | 0.46 | 0.93 |
| 9. Antibiotic resistance can develop if broad-spectrum antibiotics are used when a narrow-spectrum antibiotic would resolve the infection | 2.39 | 0.63 | 0.64 | 0.93 |
| 10. Antibiotic resistance can develop if antibiotics are used in livestock feed to promote animal growth | 2.32 | 0.67 | 0.67 | 0.93 |
| 11. Antibiotic resistance can develop if human antibiotics are used to treat infections in animals | 2.05 | 0.74 | 0.57 | 0.93 |
| 12. Antibiotic resistance can develop if antibiotics are present in human sewerage | 1.95 | 0.77 | 0.59 | 0.93 |
| 13. Antibiotic resistance can develop if antibiotics are discarded into the environment | 1.84 | 0.81 | 0.51 | 0.93 |
| 14. Resistant infections can spread from health care facilities including hospitals | 1.61 | 0.49 | 0.71 | 0.93 |
| 15. Resistant infections can spread within residential areas | 2.38 | 0.60 | 0.66 | 0.93 |
| 16. Resistant infections can spread from livestock farms | 2.32 | 0.62 | 0.69 | 0.93 |
| 17. Resistant infections can spread through wastewater | 1.27 | 0.65 | 0.67 | 0.93 |
| 18. Strict hand hygiene before and after contact with patients can help prevent the spread of antibiotic resistance between patients | 2.35 | 0.69 | 0.55 | 0.93 |
| 19. Isolation in a single room can help prevent the spread of antibiotic resistance between patients | 2.49 | 0.58 | 0.57 | 0.93 |
| 20. Appropriate environmental cleaning can help prevent the spread of antibiotic resistance between patients | 2.36 | 0.66 | 0.55 | 0.93 |
| 21. Wearing personal protective equipment such as gloves, masks and aprons can help prevent the spread of antibiotic resistance between patients | 2.51 | 0.56 | 0.65 | 0.93 |
| 22. I recognise that a person has a resistant infection when the person remains unresponsive to a number of different antibiotics | 2.50 | 0.55 | 0.59 | 0.93 |
| 23. I recognise that a person has a resistant infection by sending them for culture and sensitivity testing at a laboratory | 1.62 | 0.50 | 0.61 | 0.93 |

| Supplementary Table S3. Sources of education and information on antimicrobial resistance among family physicians (N = 351) | | |
| --- | --- | --- |
| Source of education / information | n | % of participants* |
| Undergraduate medical education (medical school courses) | 317 | 90.3 |
| In-service training (institutional seminars, continuing education programs, etc.) | 227 | 64.7 |
| Conferences, symposiums, workshops | 181 | 51.6 |
| Scientific articles and textbooks | 157 | 44.7 |
| Residency / specialty training | 148 | 42.2 |
| Information obtained from colleagues (informal education) | 146 | 41.6 |
| Training organized by professional associations | 106 | 30.2 |
| Online courses / webinars / e-learning platforms | 94 | 26.8 |
| Public health campaigns / official institutional announcements | 69 | 19.7 |
| Pharmaceutical representatives / industry-sponsored informational meetings | 51 | 14.5 |
| Media (TV, newspapers, social media, podcasts, etc.) | 51 | 14.5 |
| Training provided by international organizations (WHO, ECDC, CDC, etc.) | 30 | 8.5 |
| *Percentages are calculated based on the total number of participants (N = 351). Multiple responses were allowed; therefore, percentages do not sum to 100%. | | |
