# Supplementary material for: Antimicrobial resistance awareness and associated factors among family physicians in Turkey: a national cross-sectional study
Source: BMC Prim Care. 2026 Jun 25;27:320. doi: 10.1186/s12875-026-03444-w (PMC13523260; doi:10.1186/s12875-026-03444-w)
Supplement: Supplementary file 2 [file 12875_2026_3444_MOESM2_ESM.docx]

**Additional File 2**

**Turkish-Language Questionnaire Used in the Study**

**Note:** The questionnaire below is presented in its original Turkish-language form as administered to study participants.

**Section 1. Participant Characteristics**

1. Doğum yılınız nedir?
2. Cinsiyetinizi nasıl tanımlarsınız?

- Kadın
- Erkek
- Diğer

3. Mezuniyet yılınız nedir?
4. Şu anda görev yaptığınız ilin plaka kodu nedir?
5. Mesleki deneyim süreniz kaç yıldır?
6. Aile hekimi olarak çalıştığınız süre kaç yıldır?
7. Aile hekimliği alanında uzmanlık eğitimi aldınız mı?

- Evet
- Hayır

**Section 2. Antimicrobial Resistance (AMR)-Related Education**

8. Antibiyotik direnci ile ilgili eğitim veya bilgiyi aşağıdaki hangi kaynaklardan edindiniz? (Birden fazla seçenek işaretlenebilir)

- Üniversite eğitimi (lisans dersleri)
- Uzmanlık / ihtisas eğitimi
- Hizmet içi eğitimler (kurum içi seminer, sürekli eğitim programları vb.)
- Kongre, sempozyum, çalıştaylar
- Mesleki derneklerin düzenlediği eğitimler
- Halk sağlığı kampanyaları / resmi kurum duyuruları
- Medikal mümessiller / ilaç firması bilgilendirme toplantıları
- Çevrim içi kurslar / webinarlar / e-öğrenme platformları
- Bilimsel makale ve kitaplar
- Meslektaşlardan edinilen bilgi (informal eğitim)
- Medya (TV, gazete, sosyal medya, podcast vb.)
- Uluslararası kurumların eğitimleri (WHO, ECDC, CDC vb.)
- Diğer

9. Eğitim müfredatımın bir parçası olarak Antibiyotik Direnci hakkında bilmem gereken her şey bana öğretildi.

- Kesinlikle katılıyorum
- Katılıyorum
- Katılmıyorum
- Kesinlikle katılmıyorum

10. Antibiyotik Direnci konusunda aldığım bilgi ve eğitim günlük pratiğim için yeterlidir.

- Kesinlikle katılıyorum
- Katılıyorum
- Katılmıyorum
- Kesinlikle katılmıyorum

11. Antibiyotik Direnci ve/veya Antibiyotik Yönetimi konusunda özel bir eğitime katıldım.

- Evet
- Hayır

**Section 3. Antimicrobial Resistance Awareness Scale**

**Response options for all items in this section:**

- Kesinlikle katılıyorum
- Katılıyorum
- Katılmıyorum
- Kesinlikle katılmıyorum

**Part A. Conceptual Awareness of Antimicrobial Resistance (23 items)**

1. Antibiyotik direnci, bir mikroorganizmanın antibiyotiklere karşı dirençli hale gelmesidir.
2. Bazı mikroorganizmalar mutasyona uğrayarak antibiyotiklere direnç kazanabilir.
3. Bazı mikroorganizmalar, genetik materyal aktararak direnç özelliğini paylaşabilir.
4. Antibiyotikler, endikasyon dışında (ör. viral enfeksiyonlarda) kullanıldığında direnç gelişebilir.
5. Antibiyotik tedavisi yarıda bırakıldığında veya düzensiz kullanıldığında direnç gelişebilir.
6. Antibiyotikler önerilen dozun altında verildiğinde/kullanıldığında antibiyotik direnci gelişebilir.
7. Antibiyotikler, hastalık belirtisi olmayan taşıyıcılarda kullanıldığında direnç gelişebilir.
8. Antibiyotikler, rutin işlemler için “önlem” amaçlı kullanıldığında direnç gelişebilir.
9. Antibiyotik direnci, dar spektrumlu antibiyotik yeterli olabilecekken geniş spektrumlu antibiyotik kullanıldığında gelişebilir.
10. Antibiyotikler hayvan büyümesini teşvik etmek amacıyla yemlere katıldığında antibiyotik direnci gelişebilir.
11. İnsanlara yönelik antibiyotikler hayvan tedavisinde kullanıldığında direnç gelişebilir.
12. Antibiyotikler kanalizasyon sistemlerine karıştığında antibiyotik direnci gelişebilir.
13. Antibiyotikler çevreye atıldığında (çöp, sokak vb.) antibiyotik direnci gelişebilir.
14. Dirençli enfeksiyonlar, hastaneler de dahil olmak üzere sağlık kurumlarından yayılabilir.
15. Dirençli enfeksiyonlar toplum içinde yerleşim yerlerinde yayılabilir.
16. Dirençli enfeksiyonlar hayvancılık yapılan çiftliklerden yayılabilir.
17. Dirençli enfeksiyonlar atık su sistemleri yoluyla yayılabilir.
18. Hastalarla temas öncesi ve sonrası sıkı el hijyeni, hastalar arasında antibiyotik direncinin yayılmasını önlemeye yardımcı olabilir.
19. Hastaların tek kişilik odalarda izole edilmesi, dirençli enfeksiyonların yayılmasını önleyebilir.
20. Düzenli ve uygun çevre temizliği, hastalar arasında antibiyotik direncinin yayılmasını önleyebilir.
21. Eldiven, maske ve önlük gibi koruyucu ekipmanlar, dirençli enfeksiyonların yayılmasını önleyebilir.
22. Bir hasta farklı antibiyotiklere yanıt vermediğinde, dirençli bir enfeksiyonu olabileceği düşünülebilir.
23. Kültür ve duyarlılık testleri, dirençli enfeksiyonları tespit etmeye yardımcı olur.

**Part B. Perceptions and Prioritization of Antimicrobial Resistance (6 items)**

24. Antibiyotik reçete etmediğimde, tedavi ettiğim kişinin sağlık durumu kötüleşebilir.
25. Çalıştığım yerde, yetersiz beslenmeyi antibiyotik direncinden daha önemli bir sorun olarak görüyorum.
26. Çalıştığım yerde, kronik hastalıkları antibiyotik direncinden daha önemli bir sorun olarak görüyorum.
27. Çalıştığım yerde, hijyen ve sanitasyon düzeyini antibiyotik direncinden daha önemli bir sorun olarak görüyorum.
28. Çalıştığım yerde, diğer bulaşıcı hastalıkları antibiyotik direncinden daha önemli bir sorun olarak görüyorum.
29. Çalıştığım yerde, travma ve kazaları (örneğin trafik kazaları ve yanıklar) antibiyotik direncinden daha önemli bir sorun olarak görüyorum.

**Section 4. Awareness of Rational Drug Use Policies**

30. Sağlık Bakanlığının akılcı ilaç kullanımı uygulamalarından haberdar mısınız?

- Evet haberdarım
- Kısmen haberdarım
- Hayır haberdar değilim

31. Sağlık Bakanlığı'nın antibiyotik reçetelendirmeye yönelik performans uygulaması sonrasında reçeteleme eğiliminizde bir değişiklik oldu mu?

- Evet, daha az reçeteleme yapıyorum
- Hayır, bir değişiklik olmadı

32. Sağlık Bakanlığı'nın antibiyotik reçetelendirmeye yönelik performans uygulamasının antibiyotik direncini azaltabileceğini düşünüyor musunuz?

- Evet
- Hayır
- Fikrim yok
